# 述评：肺癌脑转移

**DOI:** 10.3779/j.issn.1009-3419.2016.08.01

**Published:** 2016-08-20

**Authors:** 孟昭 王

**Affiliations:** 100730 北京，中国医学科学院，北京协和医学院，北京协和医院呼吸内科 Department of Respiratory Disease, Peking Union Medical College Hospital, Chinese Academy of Medical Science and Peking Union Medical College, Beijing 100730, China

##  

非小细胞肺癌的中枢神经系统(central nervous system, CNS)受累是肺癌诊断和治疗中的一个重要问题, 与患者的生存期和生活质量密切相关。CNS受累包括脑转移、脑膜转移、脊髓转移、脊髓膜转移等多种形式, 患者相应的症状体征和诊断治疗均不相同。患者化疗的效果一般较差, 既往以放射治疗为主, 近期出现的分子靶向治疗、抗血管生成药物和免疫检测点抑制剂在非小细胞肺癌CNS受累的治疗中表现出来一定的疗效, 肺癌脑转移专题在相关方面均进行了涉及, 提供一些诊断和治疗的经验, 希望对广大读者有一定的帮助。综合来看, 首先要根据患者的症状体征准确及时的进行诊断; 放疗仍然是治疗的基石, 在治疗过程中始终应该考虑, 合适情况下尽早加入; 分子靶向药物有效的前提是有治疗的靶点, 因此分子靶点检测是治疗的基础; 抗血管生成药物可以提高治疗的疗效, 可以减轻血管的渗出, 缓解患者的症状; 免疫检测点抑制剂仍需要进一步积累经验。

